# Identifying Composition Novelty in Microbiome Studies: Improvement for Prediction Accuracy

**DOI:** 10.1128/mBio.00892-19

**Published:** 2019-07-30

**Authors:** Yu Sun, Yanling Li, Qianqian Yuan, Xi Fu

**Affiliations:** a Guangdong Provincial Key Laboratory of Protein Function and Regulation in Agricultural Organisms, College of Life Sciences, South China Agricultural University, Guangzhou, Guangdong, People’s Republic of China; b Key Laboratory of Zoonosis of Ministry of Agriculture and Rural Affairs, South China Agricultural University, Guangzhou, Guangdong, People’s Republic of China; c School of Public Health, Sun Yat-sen University, Guangzhou, People’s Republic of China; University of Hawaii at Manoa

## LETTER

We read with interest the paper recently published by Su et al. entitled “Identifying and Predicting Novelty in Microbiome Studies” (1). The paper presents the novel Microbiome Search Engine (MSE; http://mse.single-cell.cn/index.php/mse), which enables rapid searches of the composition similarity of query samples against sequences in Qiita (http://qiita.ucsd.edu), a well-curated and regularly updated microbiome reference database currently housing 177,022 bacterial 16S rRNA samples from humans, seawater, freshwater, soil, buildings, plants, foods, and many other environments (2, 3). Su et al. introduce a microbiome novelty score (MNS), which calculates bacterial composition difference between the query samples and the top 10 most matched microbiomes in the Qiita database. A high MNS score indicates low composition similarity to previously sampled microbiomes and high novelty. The MNS score is an easy-to-use and quantitative index to evaluate compositional uniqueness for new samples, which has the potential to be an essential analysis for future microbiome studies. However, due to technique limitation from low taxonomic resolution of 16S rRNA sequences, unexpected high similarity can be observed between unrelated samples.

**FIG 1:**
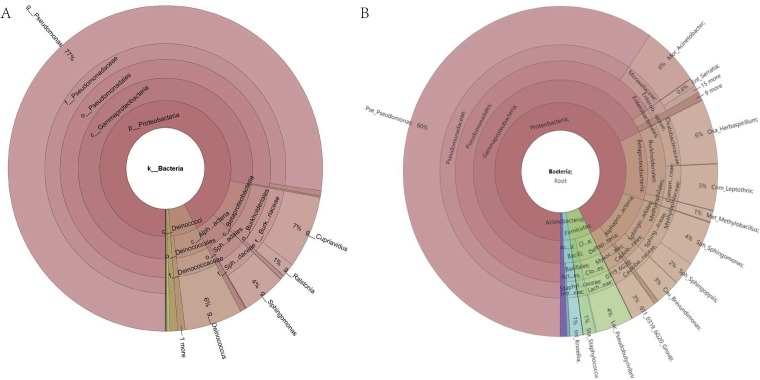
Bacterial compositions for one representative sample from a university dorm (A) and mosquito tissue (B). g_, genus; f_, family; o_, order; c_, class; p_, phylum; Alpha…acteria, *Alphaproteobacteria*; Sph…adales, *Sphingomonadales*; Sph…daceae, *Sphingomonadaceae*; Burk…riaceae, *Burkholderiaceae*; Pse_, *Pseudomonadaceae*; Mor_, *Moraxellaceae*; Ent_, *Enterobacteriaceae*; Oxa_, *Oxalobacteraceae*; Com_, *Comamonadaceae*; Met_, *Methylophilaceae*; Sph_, *Sphingomonadaceae*; Cau_, *Caulobacteraceae*; Lac_, *Lachnospiraceae*; Sta_, *Staphylococcaceae*; Int_, *Intrasporangiaceae*; Alphapro…aceteria, *Alphaproteobacteria*; Deltap…teria, Deltaproteobacteria; Myxoc…ales, *Myxococcales*; Sphingo…adales, *Sphingomonadales*; Act…es, *Actinomycetales*; Cl…es, *Clostridiales*; Caulob…rales, *Caulobacterales*; Enterb…iaceae, *Enterobacteriaceae*; Staphyl…caceae, *Staphylococcaceae*; Sping…aceae, *Sphingomonadaceae*; Comam…ceae, *Comamonadaceae*; Intr…eae, *Intrasporangiaceae*; Lach…eae, *Lachnospiraceae*; Cauloba…raceae, *Caulobacteraceae*.

We searched 83 newly sequenced settled-dust samples from dormitories in Shanxi University, China, with MSE and found that the majority of samples (67.5%) matched best with mosquito tissue samples; another 6% of samples matched birds’ egg shell sequences. The query samples also had low MNS scores (<0.09) due to the high similarities. We observed similar results in our continental hotel microbiome study, in which one sample matched mosquito sequences and one sample matched birds’ sebum sequences. Although mosquitos are present in dormitories, especially in summer, it is not possible that they contribute dominantly to this indoor environment. Many studies show that indoor microbes are mainly from outdoor environmental sources, such as air and soil, as well as human sources, such as from respiration and shedding (4–6). For both dormitory and mosquito samples, a high percentage of *Pseudomonas* spp. have been identified (Fig. 1). *Pseudomonas* is one of the most diverse and ubiquitous bacterial genera in many environments, such as sediments, water, soil, desert, plants, and animals (7). The *Pseudomonas* species in mosquito tissue certainly differs from the *Pseudomonas* sequences in dormitories, but as the 16S rRNA technique can resolve taxonomy only to the genus level, the composition similarity between the two samples is high.

To solve this issue, both the query samples and the reference database should use high-resolution taxonomic data, such as metagenomics data, to evaluate community similarity, but this approach is not economically practical for most current microbiome survey studies. Alternative solutions, such as adding a new score algorithm, can be more practical. The previous MNS score searches sequences from all environments; the new MNS score searches only a subset of environments, the query-related environments. For example, for the dormitory query samples, the new MNS score will be calculated from the building, air, soil, and other related environments, and erroneous identifications from mosquitos are thus not included. Then users can choose which MNS scores to use from their scientific perspectives.
